# Potential tactics with vitamin D and certain phytochemicals for enhancing the effectiveness of immune-checkpoint blockade therapies

**DOI:** 10.37349/etat.2023.00145

**Published:** 2023-06-30

**Authors:** Ai Tsuji, Sayuri Yoshikawa, Sae Morikawa, Yuka Ikeda, Kurumi Taniguchi, Haruka Sawamura, Tomoko Asai, Satoru Matsuda

**Affiliations:** Istituto Nazionale Tumori-IRCCS-Fondazione G. Pascale, Italy; Department of Food Science and Nutrition, Nara Women’s University, Kitauoya-Nishimachi, Nara 630-8506, Japan

**Keywords:** Vitamin D, programmed cell death-1 (PD-1), cytotoxic T-lymphocyte-associated protein-4 (CTLA-4), immune checkpoint, estrogen, peroxisome proliferator-activated receptor (PPAR), phosphoinositide-3 kinase (PI3K)/AKT, cancer therapy

## Abstract

Immunotherapy strategies targeting immune checkpoint molecules such as programmed cell death-1 (PD-1) and cytotoxic T-lymphocyte-associated protein-4 (CTLA-4) are revolutionizing oncology. However, its effectiveness is limited in part due to the loss of effector cytotoxic T lymphocytes. Interestingly, supplementation of vitamin D could abolish the repressive effect of programmed cell death-ligand 1 (PD-L1) on CD8^+^ T cells, which might prevent the lymphocytopenia. In addition, vitamin D signaling could contribute to the differentiation of T-regulatory (Treg) cells associated with the expression of Treg markers such as forkhead box P3 (FOXP3) and CTLA-4. Furthermore, vitamin D may be associated with the stimulation of innate immunity. Peroxisome proliferator-activated receptor (PPAR) and estrogen receptor (ESR) signaling, and even the signaling from phosphoinositide-3 kinase (PI3K)/AKT pathway could have inhibitory roles in carcinogenesis possibly via the modulation of immune checkpoint molecules. In some cases, certain small molecules including vitamin D could be a novel therapeutic modality with a promising potential for the better performance of immune checkpoint blockade cancer therapies.

## Introduction

**Figure 1:**
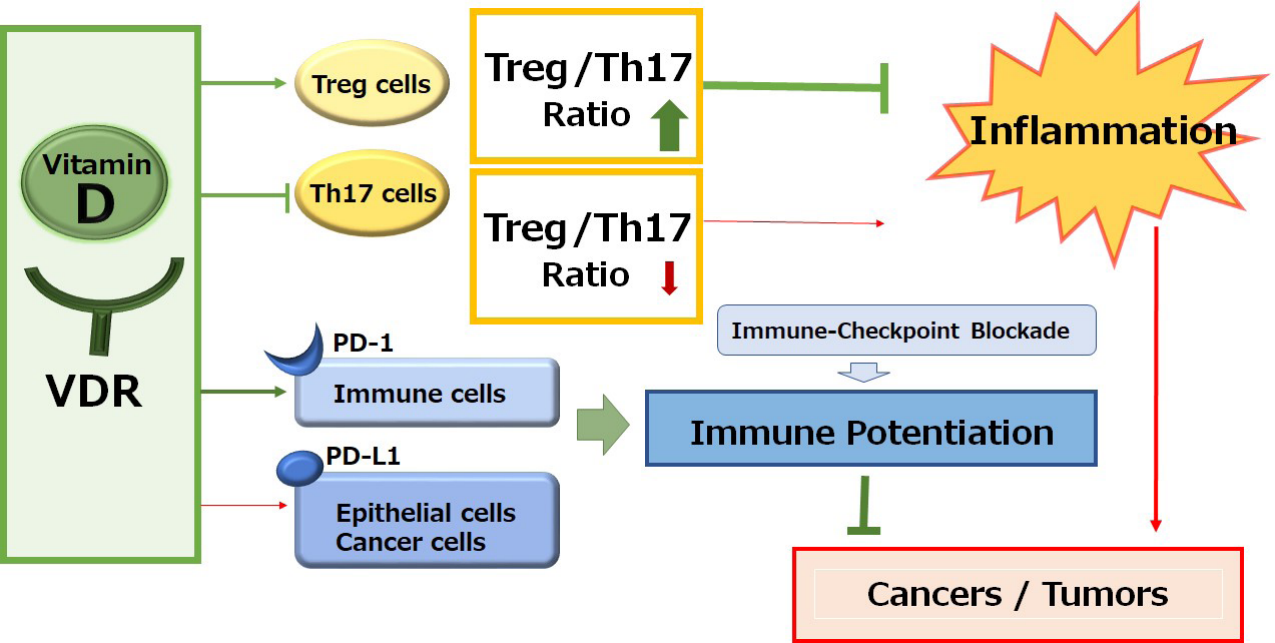
A schematic representation and overview of the vitamin D signaling pathway for the immunological alteration. Through the activation of vitamin D and its receptor (VDR) signaling, the improved Treg/Th17 cells balance could contribute to the inhibition of inflammation. On the other hand, the activation of vitamin D and VDR signaling could also alter the expression of immune checkpoint molecules for the improvement of immune checkpoint blockade therapies. Arrowhead indicates stimulation whereas hammerhead shows inhibition. Note that several important activities such as cytokine-induction or anti-inflammatory reaction have been omitted for clarity

Immune checkpoint inhibitors targeting the signaling pathway of immune checkpoint molecules such as programmed cell death-1 (PD-1)/programmed cell death-ligand 1 (PD-L1) have improved the prognosis for various malignancies [1]. The anti-tumor activity of these inhibitors results from intensification of the T cell immune response, which could also protect against harmful inflammation and/or autoimmunity [2]. Basically, humanized monoclonal antibodies to immune checkpoints could stimulate T cells and/or discharge the immunity from distinguishing cancer cells. Effective anti-tumor immune responses may have a need for CD8^+^ and CD4^+^ T cells [3, 4]. Owing to the disappointing efficacy of immune blockade cancer therapy, the immunotherapy might occasionally combine with chemotherapy and/or radiotherapy. These combination cancer therapies have been approved as fruitful first-line therapy for various malignant cancers [5]. However, the efficacy of PD-1 blockade cancer immunotherapy may be limited due to the loss of effector cytotoxic T lymphocytes. Besides, even though immunotherapy by PD-1 blockade has radically improved the survival rate of the patients with malignancy, further improvement in efficacy may be required for reducing the percentage of less sensitive patients. Therefore, exploring the mechanisms of insensitivity to the immune blockade cancer therapy has appeared as one of the most imperative tasks in developing more effective cancer therapy. It is well known that vitamin D deficiency decreases the numbers of CD4^+^ and/or CD8^+^ T lymphocytes, whereas the supplementation of vitamin D increases CD4^+^ lymphocytes. In addition, vitamin D can activate T-regulatory (Treg) cells, which could repress proinflammatory responses [6]. Likewise, vitamin D could make the cancer microenvironment uncomfortable for cancer cells by increasing the ratio of Treg/T-helper 17 (Th17) cells (Figure 1) [7, 8]. Consistently, vitamin D deficiency and/or genetic polymorphisms in genes involved in the vitamin D metabolism might be related to the higher risk of several autoimmune diseases and/or cancers [9]. In addition, it has been shown that vitamin D could induce the expression of PD-L1 on gut epithelial cells and that of PD-1 on immune cells in patients with inflammatory bowel diseases such as ulcerative colitis (Figure 1) [10].

Vitamin D is a steroid hormone with the main function of regulating bone metabolism, but with many other physiological functions, such as anti-inflammatory, immunomodulatory, and anti-angiogenic effects, theoretically acting as a carcinogenesis inhibitor [11]. The value of vitamin D may be also recognized as a protective agent against several cancers [12]. It is shown that an increase in vitamin D intake could decrease the risk of some cancers [13]. Vitamin D could modulate tumor growth and inflammation in the tumor microenvironment, however, which might be reversed in high-fat diet conditions, suggesting the importance of diet on tumor growth [14]. Epidemiological studies also suggest that vitamin D deficiency increases the incidence of colorectal cancer, and that it has a negative impact on survival of the patients with colorectal cancer [15]. Levels of active vitamin D have been downregulated in the serum of patients with colorectal cancer, which might be correlated with increased Th17 lymphocytes [16]. Accordingly, vitamin D might play a protective and beneficial role in cancer survival [17]. In fact, vitamin D supplementation has been associated with a reduction in lung cancer mortality [18]. Vitamin D might also play a role of prevention in colorectal cancer [16]. In general, vitamin D is the principal mediator of the beneficial effects of sun exposure [19]. Food sources of vitamin D include mushrooms, eggs, salmon, eel, tuna, yogurt, and milk [20]. Mechanistically, the active metabolite form of vitamin D applies to the cells through binding to VDR [21]. The small intestine is the organ showing the highest expression of VDR. It may exert anti-proliferative effects by inducing cell cycle arrest and/or by inducing apoptosis. In addition, vitamin D exerts an anti-inflammatory effect by acting as a potent inhibitor of tumor cell-induced angiogenesis and/or inhibiting stress-activated kinase signaling. However, overexpression of *VDR* gene has been significantly associated with worse survival in breast cancer [22]. Vitamin D or its analogs could affect multiple biochemical pathways, which may modulate pathophysiological mechanisms including the carcinogenesis [23]. It has been suggested that the influence of vitamin D on Th17 lymphocytes may be one of the mechanisms supporting tumor metastasis in mice models [24]. Researchers have recently begun to investigate the inhibitory effects of dietary vitamin D on cancer stem cells [25]. In this review, we would like to discuss and summarize the therapeutic impact of vitamin D and/or its molecular processes to target cancer cells.

## Connection between vitamin D and immune cells

Vitamin D, which here means general types of all vitamin D including vitamin D_2_, vitamin D_3_, 25(OH)D_3_, and/or 1,25(OH)2D_3_ for clarity and readability, may be associated with the stimulation of innate immunity, inflammation, and host defense against pathogens [26]. In fact, an essential role of vitamin D has been suggested in macrophage differentiation that could modulate host response against pathogens, inflammation, and cellular stresses [26]. In addition, pathogen-informed dendritic cells (DCs) could provoke Th17 cells from memory T cells [27], which could progress the recruitment of neutrophils to the inflammatory spot [28], with making and/or enhancing an inflammatory loop. Therefore, the restriction of Th17 cells expansion might be favorable in the treatment of several immune-related diseases. Vitamin D might be an actual anti-inflammatory molecule that could work for the prevention and/or the treatment of autoimmune diseases [29]. Furthermore, VDR is highly expressed within Th17 lymphocytes. Active vitamin D can decrease the recruitment of Th17 cells through the VDR-mediated pathway [30]. Furthermore, an inflammatory situation that brings the inappropriate ratio of Th17/Treg cells could be controlled by vitamin D [31]. Vitamin D can regulate the expression of several genes linked to Th17 cells and Treg cells, lessening the fraction of Th17 cells while growing the proportion of Treg cells (Figure 1) [32]. It has also been shown that vitamin D administration could elevate the levels of Th1 and Treg cells, whereas the level of Th2 and Th17 might be diminished [33]. Similarly, vitamin D might have anti-inflammatory potential in the treatment of the ulcerative colitis via the reduction of Th17 cells. [34]. Vitamin D is a familiar regulator of immune responses, acting on several immune cell types, including T cells, B cells, macrophages, antigen-presenting cells, and DCs, which all express VDR [35]. Adequate levels of vitamin D are recommended to keep good immunity and/or prevent various immunological disorders including autoimmune diseases. Nonetheless, vitamin D deficiency may be likely to occur as a result of combined factors such as poor/inadequate diet and sun underexposure [36].

Remarkably, vitamin D could also support immunity, not only acting directly on immune cells but also modulating the other conventional immune tissues such as the skeletal muscle [37]. In addition to the resident immune compartments, the skeletal muscle seems to act as a suitable immune modulatory organ. For example, vitamin D could play an important role in maintaining a healthy mineralized skeleton, which may be also considered as an immunomodulatory organ for the regulation of innate and/or adaptive immune systems [38]. The immunological function within skeletal muscle recognized as an organ with immune capacity might be under the tight control of vitamin D [39]. Vitamin D has been found to improve the intestinal microbiome, immune system, and facilitate muscle anabolism [40]. Therefore, the interplay between exercise and vitamin D status could play a pivotal role in immune and/or health homeostasis.

## Connection between vitamin D and cancer related immunity

Many studies have established the unsuitable presence of Th17 cells in various types of cancer such as colorectal, breast, and ovarian cancers [41]. However, it is challenging to define clear roles of the Th17 cell in tumor development due to the intricate interaction between cancer cells and the cancer microenvironment [42]. Inflammation has been shown to be frequently associated with cancer progression, which could contribute to the survival, angiogenesis, and metastasis of cancer cells [43]. It is recognized that tumor cells could build an inflammatory environment advantageous for the recruitment of Th17 cells [44]. In general, proinflammatory activities could be inhibited by vitamin D by suppressing proinflammatory cytokines [45]. Expression of vitamin D-related enzymes as well as *VDR* gene polymorphism has been suggested in various stages of cancer development [46]. Vitamin D could regulate the angiogenesis that is involved in metastasis by controlling the expression of adhesion molecules in cancer cells [47], indicating that higher levels of serum vitamin D are associated with better prognosis. In fact, patients with advanced metastatic breast cancer have shown meaningfully lower levels of vitamin D than patients with early-stage disease [48, 49]. Similarly, decreased levels of VDR expression in tumor has been associated with aggressive appearances of breast cancers [50]. Additionally, the VDR expression in circulating tumor cells, which could be easily detected, has been proposed as a prognostic biomarker for breast cancers [51].

**Figure 2:**
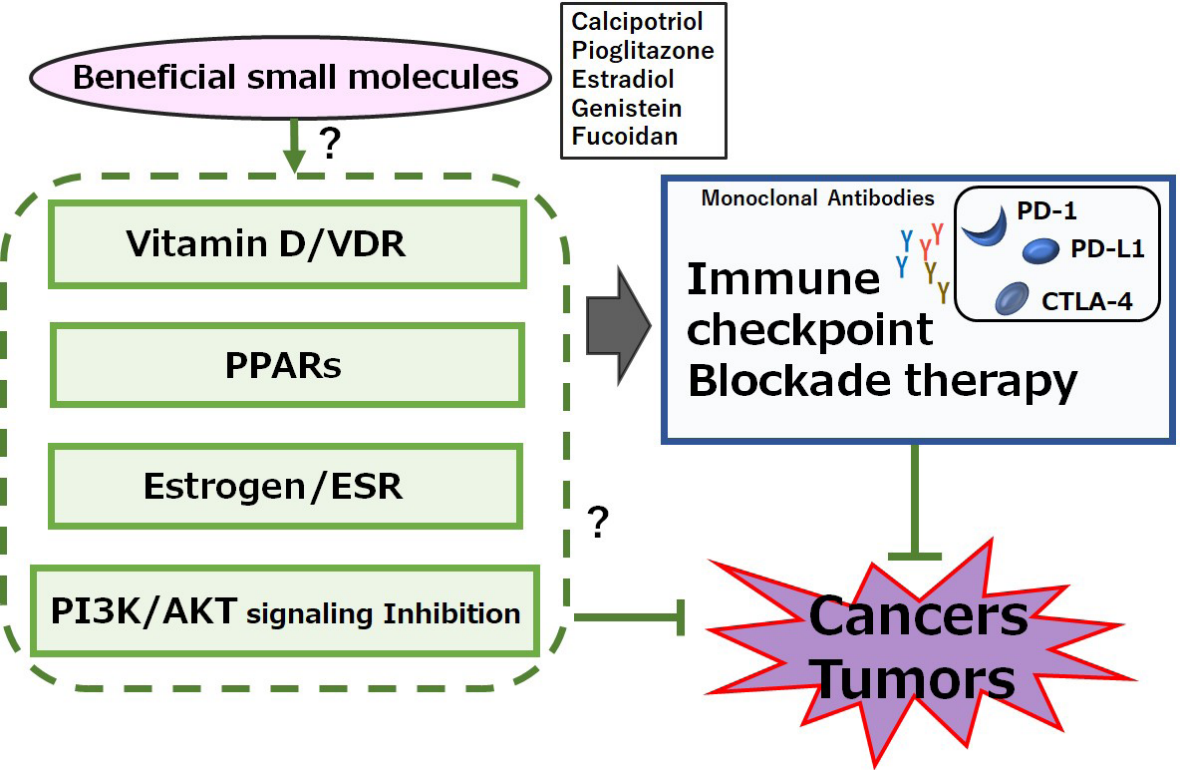
A possible beneficial mechanisms of some small molecules for cancer therapy. A hypothetical schematic representation and overview suggesting that immune checkpoint blockade therapies with monoclonal antibodies against PD-1, PD-L1, or CTLA-4 might be potentiated by several signaling pathways including VDR, peroxisome proliferator-activated receptor (PPAR), estrogen receptor (ESR), and/or phosphoinositide-3 kinase (PI3K)/AKT pathway. Certain small molecules that could activate the signaling pathway might also have a potential to enhance the efficacy of immune checkpoint blockade therapies. Examples of certain beneficial small molecules with some effects on anti-cancer responses have been shown on the right side of “certain small molecules”. Arrowhead indicates stimulation whereas hammerhead shows inhibition. Note that several important activities have been omitted for clarity

Calcipotriol, a synthetic vitamin D analog, can activate CD8^+^ T lymphocytes with a concomitant reduction in the number of Treg cells in glioblastoma multiforme, which can be a novel therapeutic modality to overcome the immune resistance of glioblastoma multiforme by converting immunologically “cold” tumors into “hot” tumors [52]. It has been shown that the CD8^+^ tumor-infiltrating T cells are associated with improved survival in triple-negative breast cancer [53]. In these ways, vitamin D could modulate tumor growth by the alteration of T lymphocytes balance in the tumor microenvironment (Figure 1). In addition, vitamin D could mediate immunomodulating activities [54], which have been widely explored in autoimmune disorders including inhibition of Th lymphocytes. Serum levels of vitamin D are negatively correlated with expression of PD-1, which could enhance the antitumor immunity [55]. The poor outcome with PD-1 blockage immunotherapy may be at least in part mediated by vitamin D deficiency-induced impairment of immune function [56]. Interestingly, the supplementation of vitamin D could remove the suppressive effect of PD-L1 on CD8^+^ T cells, consequently preventing lymphopenia and reducing disease mortality and/or severity in patients with coronavirus disease 2019 (COVID-19) [57]. Therefore, adequate levels of serum vitamin D are required in order to maintain optimal immune surveillance even against cancers [58]. Interestingly, it has been shown that increased levels of five soluble inhibitory immune checkpoint molecules including cytotoxic T-lymphocyte-associated protein-4 (CTLA-4), PD-1, PD-L1, lymphocyte-activation gene-3 (LAG-3), and T cell immunoglobulin and mucin-domain containing-3 (TIM-3) with the setting of decreased vitamin D in xeroderma pigmentosum (XP) patients suggest a possible role of ongoing immune suppression in the pathogenesis of XP-associated malignancies [59]. Particularly, systemic concentrations of CTLA-4 and PD-1 might be considerably increased in basal cell carcinoma [60]. In addition, vitamin D signaling via VDR could contribute to Treg cells differentiation, while also being positively associated with the expression of key Treg marker including forkhead box P3 (FOXP3) and CTLA4 [61]. There is a significant relationship between high VDR expression in cancer cells and low CTLA-4 expression with favorable prognostic parameters such as low stage of tumors and/or invasions (Figure 2) [62].

## Synergistic effect between PPAR, ESR, and VDR

PPAR may have also anti-tumor actions in colon, breast, prostate, and lung cancers [63]. Noteworthy, remarkable redundancy may exist between PPAR and the vitamin D/VDR system regarding its protective role in carcinogenesis [47, 64]. The vitamin D signaling is likewise susceptible to epigenetic regulation [65]. Interestingly, this epigenetic repression of vitamin D is almost always existing in cancers [66], which might fascinatingly contribute to the possibility of the same phenomena occurring in the PPARγ signaling. Remarkably, potent vitamin D response elements have been revealed in human PPARδ promoter [67], which might be one evidence that epigenetic events could affect both on PPARγ and on vitamin D/VDR signaling. However, some studies have shown the ability of PPARγ to bind VDR and inhibit vitamin D-mediated transactivation [68]. The PPARγ could also modulate PD‐1 expression [69]. An agonists of peroxisome PPARγ, bezafibrate, could increase cytotoxic T lymphocytes by stimulating mitochondrial metabolism, subsequently leading to the greater antitumor immunity during PD-1 blockade [70]. The activation of PPARγ could also enhance Treg cells response, which is beneficial for inflammatory diseases [71]. PPARγ is an intrinsic suppressor for Th17 cells generation, which could contribute to the possibilities for specific immunointervention in Th17-mediated diseases [72]. In addition, it has been reported that PPARα deficiency might exert anti-cancer properties by diminishing the function of Treg cells and/or upregulating pro-inflammatory T cells [73].

Activity of estrogen might be mediated by its binding to ESRs which are categorized as a nuclear receptor superfamily of transcription factors. ESRs could further bind to the estrogen response element (ERE) existing at the promoter region of target genes [74]. ESRs are composed of ESR1 and ESR2 encoded by *ESR1* and *ESR2* genes, respectively. Therefore, estrogen-dependent breast cancers predominantly express ESR1 or ESR2, which could basically transduce the activity of estrogens [75]. Interestingly, there is evidence of synergistic effect between estrogen and vitamin D. For example, it has been revealed that vitamin D could stimulate the production of estrogens which are indispensable for the function of VDR in the central nervous system [76]. In mouse spleen cells, estrogen could inhibit Th17 cells differentiation [77]. Th17 cells express both ESR1 and ESR2. Similarly, estrogen could increase and/or differentiate the Th17 cells accompanied by downregulation of FOXP3 in estrogen deficiency-induced bone loss [78]. In addition, estradiol treatment may increase the number of Th17 cells during the development of arthritis [79]. Other studies have revealed that ESR1 signaling may increase interleukin-17 (IL-17) production in Th17 cells by supporting mitochondrial proliferation [80]. Therefore, deletion of ESR1, but not ESR2, has triggered a significant reduction in the production of IL-17A on Th17 cells. Consequently, the ESR1 signaling could regulate Th17 cell differentiation [81]. Thus, it appears that the impact of estrogen on Th17 cells might be determined by the environment. Interestingly, high levels of ESR expression could repress the infiltration of Th17 and/or CD8^+^ T cells by causing a reduction of PD-1/PD-L1 expression in breast cancers (Figure 2) [82].

## VDR, PPAR, and ER involved in carcinogenesis

The above-mentioned inhibitory roles of VDR, PPAR, and ESR in carcinogenesis are probably dependent on the cellular context, cell type, differentiation stage, and the microenvironment of cells. It has been reported that hypo-vitaminosis D is associated with various chronic diseases including cancer [11, 83]. In addition, the association between vitamin D deficiency and carcinogenesis had been considered [84]. However, it is now recognized that vitamin D also affects the processes of cell proliferation potentially leading to carcinogenesis [85]. These mechanistic relationships between vitamin D and cancer have been deeply focused [86]. Although many epidemiological studies have shown that the vitamin D levels in serum are not likely related to the risk of developing cancer, it has been found by a meta-analysis that the vitamin D levels may be related to morbidity and mortality outcomes in patients with cancer [87].

PPAR family is also known to be engaged in a variety of biological processes, including carcinogenesis [88]. In fact, PPARγ plays a crucial role in the metabolic reprogramming of cancer-associated fibroblasts and adipocytes, occasionally driving to become substrate donors for cancer growth [89]. Overexpressed PPARs have been observed in many human cancers [90]. Moreover, the increase in its overexpression of PPARs may be correlated with poor survival of patients with various types of cancers [90]. Furthermore, estrogen might stimulate the development of predominant breast cancers [91]. In particular, ESR signaling is a key driver of ER^+^ breast carcinogenesis [92]. ESRs are all interrelated to an increased risk of breast cancers in a prospective study [93, 94]. A low-fat diet and a vegetarian diet could reduce levels of sex-steroid hormones and the risk of breast cancers [95], suggesting that dietary and environmental factors may be responsible for the incidence of breast cancers [96]. On the other hand, phytoestrogen genistein, a soybean isoflavone, may act as an estrogen agonist on human ESRs [97]. However, genistein could induce apoptosis in breast cancers even with ESR-negative cells, suggesting that the growth-inhibitory effects of genistein might be via the estrogen-independent signaling pathways [97].

When, how, and why do their contradictory effects of VDR, PPAR, and ESR on cancer cells might occur? Although the precise mechanisms have not been elucidated yet, a reasonable explanation has been suggested in the similar inconsistency with anti-proliferative proteins, in which the roles of certain exosomes, matrix metalloproteinases (MMPs), and microRNAs might be involved [98].

## Future perspectives

The PI3K/AKT signaling may be involved in the processes of cancer cells’ growth and/or apoptosis. In addition, T cells proliferation and/or migration may be also regulated by the PI3K/AKT signaling pathway. Moreover, it has been revealed that Th17 cell differentiation can be regulated by mammalian/mechanistic target of rapamycin (mTOR) complex 1 (mTORC1) via the PI3K/AKT pathway [99]. While stimulation of PI3K and/or mTORC1 signaling could enhance the differentiation of Th17 cells, the inhibition of PI3K and/or mTORC1 in CD4^+^ T cells could initiate the differentiation of Treg cells [100]. Therefore, several activators of PI3K/AKT, adenosine monophosphate-activated protein kinase (AMPK), and mTOR could synergize the immune checkpoint blockade therapy, suggesting a way to develop novel combinatorial therapies with immune checkpoint blockade [101]. In fact, mTOR inhibitors such as rapamycin could decrease the expression of PD-L1 [102], while enhanced expressions of AKT and/or mTOR could increase the PD-L1 expression [103]. Similarly, abnormal activation of PI3K/AKT/mTOR pathway may also result in increased translation of PD-L1 protein, suggesting that combining therapy with immune checkpoint blockades and PI3K/AKT/mTOR inhibitors could extend and/or enhance the cancer therapies (Figure 2) [104]. Interestingly, a low molecular weight fucoidan could inhibit the PI3K/AKT signaling pathways [105]. In addition, dexmedetomidine, a highly selective agonist of the α2-adrenergic receptor, is clinically used for the sedation of patients, which could reduce the expression of phosphorylated PI3K and phosphorylated AKT [106]. Furthermore, an imidazo pyridine derivative has proved to be a potent PI3K/mTOR dual inhibitor with excellent kinase selectivity, modest plasma clearance, and acceptable oral bioavailability [107]. It might be noted that the PI3K/AKT/mTOR signaling may be in one of the main signaling pathways involved in carcinogenesis and metastasis [108]. As shown here, the role of vitamin D could be involved in modulating the levels of immune-checkpoint molecules, probably via VDR, PPAR, ESR, and/or the PI3K/AKT/mTOR pathways, to enhance their anticancer efficacy. However, the negative impacts of vitamin D on DCs and B cells have been suggested [24, 109]. In some cases, vitamin D has immunosuppressive properties, which may adversely affect the efficacy of cancer therapies [110]. Therefore, it is compulsory to investigate in-depth the action of vitamin D, which might contribute to the development for the intervention against tumor formation and metastasis. Also, more attention should be paid to several nuclear receptors including VDR, PPAR, and ESR expressed within tumor cells as well as the activation of PI3K/AKT/mTOR pathways on some stages of tumors in order to make some information useful in customizing the superior personalized cancer treatment.

## Conclusion

Vitamin D could inhibit the progression of quite a few tumors by several mechanisms, which might be a booster for the better performance of several immune checkpoint therapies. Depending on how to use it, it must be a promising option for the improvement of cancer therapies.

## Abbreviations

CTLA-4: cytotoxic T-lymphocyte-associated protein-4
DCs: dendritic cells
ESR: estrogen receptor
mTOR: mammalian/mechanistic target of rapamycin
mTORC1: mammalian/mechanistic target of rapamycin complex 1
PD-1: programmed cell death-1
PD-L1: programmed cell death-ligand 1
PI3K: phosphoinositide-3 kinase
PPAR: peroxisome proliferator-activated receptor
Th17: T-helper 17
Treg: T-regulatory
VDR: vitamin D receptor

## Declarations

### Copyright

© The Author(s) 2023.
